# High magnetoresistance of a hexagonal boron nitride–graphene heterostructure-based MTJ through excited-electron transmission†

**DOI:** 10.1039/d1na00272d

**Published:** 2021-10-21

**Authors:** Halimah Harfah, Yusuf Wicaksono, Gagus Ketut Sunnardianto, Muhammad Aziz Majidi, Koichi Kusakabe

**Affiliations:** Graduate School of Engineering Science, Osaka University 1-3 Machikaneyama-cho Toyonaka Osaka 560-8531 Japan harfah.h@opt.mp.es.osaka-u.ac.jp; Department of Physics, Faculty of Mathematics and Natural Science, Universitas Indonesia Kampus UI Depok Depok Jawa Barat 16424 Indonesia; Research Center for Physics, The National Research and Innovation Agency Kawasan Puspiptek Serpong Tangerang Selatan Banten 15314 Indonesia; School of Science, Graduate School of Science, University of Hyogo 3-2-1 Kouto, Kamigori-cho, Ako-gun Hyogo 678-1297 Japan

## Abstract

This work presents an *ab initio* study of a few-layer hexagonal boron nitride (hBN) and hBN–graphene heterostructure sandwiched between Ni(111) layers. The aim of this study is to understand the electron transmission process through the interface. Spin-polarized density functional theory calculations and transmission probability calculations were conducted on Ni(111)/*n*hBN/Ni(111) with *n* = 2, 3, 4, and 5 as well as on Ni(111)/hBN–Gr–hBN/Ni(111). Slabs with magnetic alignment in an anti-parallel configuration (APC) and parallel configuration (PC) were considered. The pd-hybridizations at both the upper and lower interfaces between the Ni slabs and hBN were found to stabilize the system. The Ni/*n*hBN/Ni magnetic tunnel junction (MTJ) was found to exhibit a high tunneling magnetoresistance (TMR) ratio at ∼0.28 eV for *n* = 2 and 0.34 eV for *n* > 2, which are slightly higher than the Fermi energy. The observed shifting of this high TMR ratio originates from the transmission of electrons through the surface states of the d_*z*^2^_-orbital of Ni atoms at interfaces which are hybridized with the p_*z*_-orbital of N atoms. In the case of *n* > 2, the proximity effect causes an evanescent wave, contributing to decreasing transmission probability but increasing the TMR ratio. However, the TMR ratio, as well as transmission probability, was found to be increased upon replacing the unhybridized hBN layer of the Ni/3hBN/Ni MTJ with graphene, thus yielding Ni/hBN–Gr–hBN/Ni. A TMR ratio as high as ∼1200% was observed at an energy of 0.34 eV, which is higher than the Fermi energy. Furthermore, a design is proposed for a device based on a new reading mechanism using the high TMR ratio observed just above the Fermi energy level.

## Abstract 2

This work presents an *ab initio* study of a few-layer hexagonal boron nitride (hBN) and hBN–graphene heterostructure sandwiched between Ni(111) layers.

## Graphical abstract

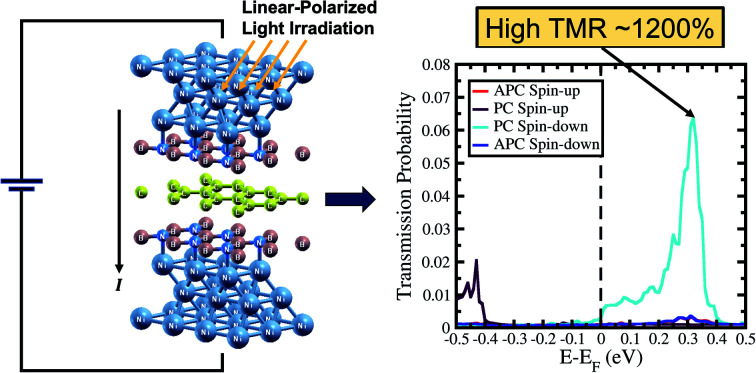

## 1 Introduction

Magnetic tunnel junctions (MTJs) are one of the most important devices for spintronic applications. Many studies of MTJs have been undertaken,^1–13^ indicating the potential of the devices' application to logic devices, hard-drive magnetic read heads, and magnetic sensors.^14–19^ The primary competition for improvement of MTJs is to attain a higher percentage of the tunneling magnetoresistance (TMR) ratio. One of the vital points is their tunnel barrier. The common tunnel barrier used is MgO. Among various MTJs, CoFeB/MgO/CoFeB showed the highest TMR ratio of 1100% at 4.2 K.^20^ In order to downscale the device, retaining a high transmission and reducing the barrier thickness are essential. However, when this is realized, TMR ratio can be reduced to 55% due to the presence of uncontrollable defects in the MgO tunnel barrier.^21^

Recently, 2D materials have been examined for nonmagnetic spacers in current-perpendicular-to-plane (CPP) MTJs. Currently, the performance on the TMR ratio is still relatively low.^22^ However, one-atom-thick materials, such as graphene and hexagonal Boron Nitride (hBN), owing to their extraordinary properties, are expected to impart a unique characteristic to MTJs and enable a new device mechanism that could not be found in conventional ones. The chemical and physical interactions between graphene or hBN and the ferromagnetic layer are interesting and can be further developed. For instance, when graphene is realized as a tunnel barrier of MTJs, small magnetoresistance (MR) was found when the CPP is considered.^2,10,11,22–29^ However, a recent theoretical study suggested that when the Ni/graphene/Ni system is considered, a controllable gapped-Dirac cone can be realized, leading to a high current-in-plane (CIP) magnetoresistance ratio.^30^ A similar case is also found in our previous study on monolayer hBN. A novel functionality of spin-current control through cross-correlation properties of the bi-stable state at Ni/hBN/Ni junction interfaces was recently discovered, which could not be obtained in conventional MTJs.^31^

A van der Waals heterostructure of graphene–hBN has recently attracted huge interest due to its unique properties. Fabrication of the graphene–hBN van der Waals heterostructure has been further developed, and creation of the graphene–hBN heterostructure has become possible.^32^ A previous experimental study shows the use of a graphene–hBN heterostructure as a tunnel barrier of 2D material based MTJs and found an increasing performance compared with that of graphene or hBN based MTJs, and a unique inverted signal of the spin valve has been found.^33^ The unique chemical and physical properties are expected between the interface and insulator barrier. Therefore, a further theoretical investigation is required to attain a full understanding of the MTJ properties.

This paper presents a comprehensive theoretical study on a Ni/hBN–Gr–hBN/Ni MTJ. It was found that this MTJ exhibits a TMR ratio as high as ∼1200% at energy slightly higher than Fermi energy, and it is envisaged that this material system will have a new reading process mechanism. To understand the unique properties at the interfaces of the Ni/hBN–Gr–hBN/Ni MTJ, an investigation was also conducted on Ni/*n*hBN/Ni MTJs with *n* = 2, 3, 4, and 5. Also, in this case, it was found that the high TMR ratio does not occur at the Fermi energy level but rather at slightly higher energy, namely 0.28 eV for *n* = 2 and 0.34 eV for *n* > 2. The shifting of the high TMR ratio originates from the transmission of electrons through the surface state of the d_*z*^2^_-orbital of the Ni atoms at the interfaces, which hybridize with the p_*z*_-orbital of the N atoms. When *n* > 2 is considered, a proximity effect occurs, resulting in an evanescent wave and contributing to the change of transmission probability, leading to a change in the TMR ratio. The evanescent wave still exists at *E* − *E*_F_ = 0.34 eV but is relatively weak due to the proximity effect of the d_*z*^2^_-orbital of Ni on the p_*z*_-orbital of boron, which is unoccupied. The role of the graphene layer in the Ni/hBN–Gr–hBN/Ni MTJ becomes a key point to strengthen the proximity effect of the d_*z*^2^_-orbital inside the insulator barrier, resulting in high TMR ratio.

## 2 Computational method

Spin-polarized plane wave-based density functional theory (DFT) calculations were performed using the Quantum ESPRESSO package.^34,35^ A revised Perdew–Burke–Ernzerhof (PBE) functional for a densely packed solid surface, called the PBESol functional,^36^ as well as ultrasoft pseudopotentials,^37^ within the generalized gradient approximation (GGA) was used to describe the electron–ion interaction. A kinetic energy cut-off of 50 Ry was used for the wavefunctions to reach a good convergence calculation. Since adopting an appropriate *k*-point grid can result in the convergence of the total energy calculation in this system, a *k*-point grid of 24 × 24 × 1 was chosen for all calculations. Furthermore, the effect of the van der Waals interactions on the electronic structures was included by applying the most recent and accurate DFT-D3 corrections.^38^ Taking into account the van der Waals interactions in the DFT calculations, the average interlayer distances of Ni–hBN and hBN–hBN are 2.05 Å and 3.13 Å, respectively. These results are close to the experimental values, which are 1.87 ± 0.12 Å for the Ni–hBN distance in the hBN/Ni system^39^ and 3.33 Å for the hBN–hBN distance in the hBN bulk.^40^

**Fig. 1:**
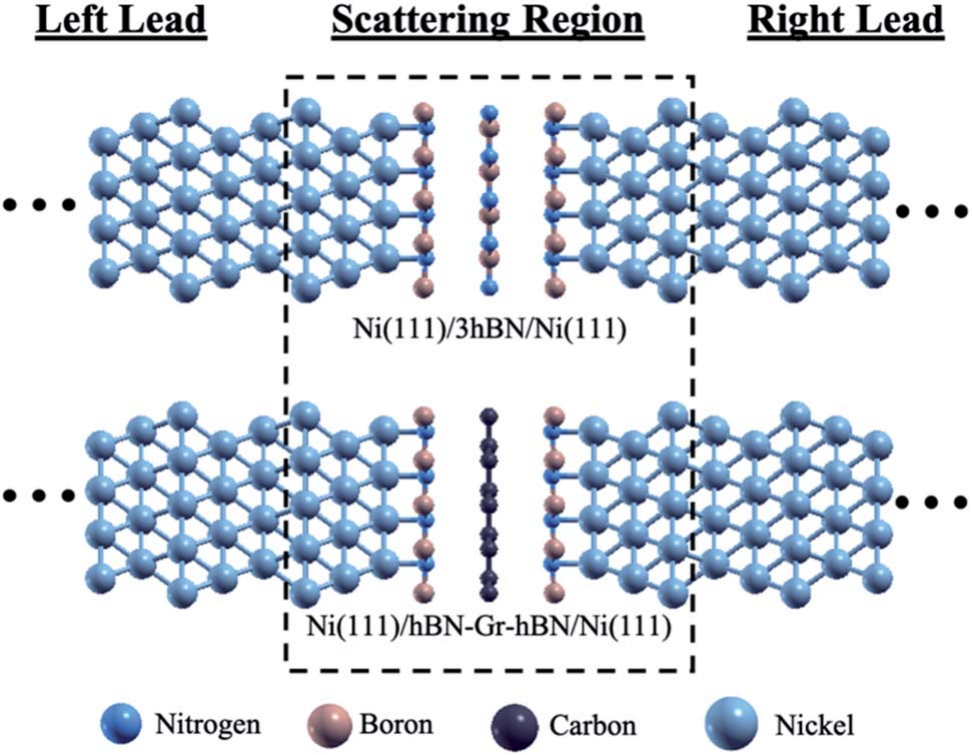
Side view of the supercell of Ni(111)/3hBN/Ni(111) (as a representative of a few-layer hBN MTJ) and Ni(111)/hBN–Gr–hBN/Ni(111) used to represent the scattering region and lead corresponding to the model calculation for transmission probability calculation.

The ballistic transmission probability calculations were performed using the Landauer–Buttiker formalism.^41,42^ The left lead, right lead, and scatterer regions were considered in the model calculation, as shown in Fig. 1. Ni(111) was considered for the left and right leads to reduce the computational cost without neglecting any important physics. However, the use of Au(111) as a lead carrying normal input/output currents for the Ni/*n*hBN/Ni and Ni/hBN–Gr–hBN/Ni structures would be expected in an actual device. The current flow from the left to the right lead through the scatterer can be expressed as follows:1
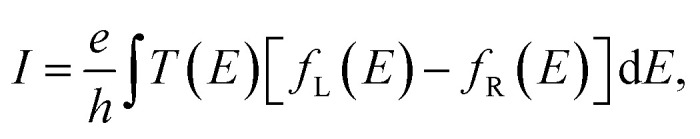
where *f*_L_(*E*) (*f*_R_(*E*)) is the right-(left-)moving electrons injected from the left (right) lead in the form of Fermi–Dirac function, respectively. Additionally, the ballistic transmission *T* as a function of the energy *E* is described as2
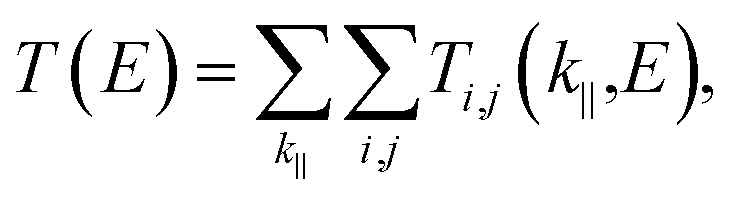
where *T*_*i*,*j*_ (*k*_‖_,*E*) is the probability of electrons with energy *E* and state. *T*(*E*) is then obtained by summing over the 2D Brillouin momentum *k* to move from the *i*-th Bloch state to the *j*-th Bloch zone and over all incoming–outgoing states. In the present study, the transmission probability calculations were performed using the PWCOND^43^ module of the Quantum ESPRESSO software. A perpendicular *k*-point of 50 × 50 with respect to the transmission direction is considered to get a good accuracy of transmission probability.

The PWCOND module calculates the transmission probability at zero temperature from which the conductance of the system can be calculated as follows:3
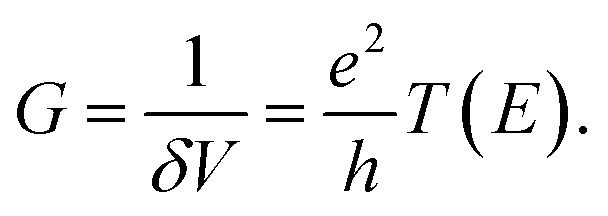


Finally, a magnetoresistance ratio calculation was conducted based on the Julliere model.^44^ The calculation was done by including the difference between the conductance in the APC and PC states and then dividing it by the conductance in the APC state, according to:4
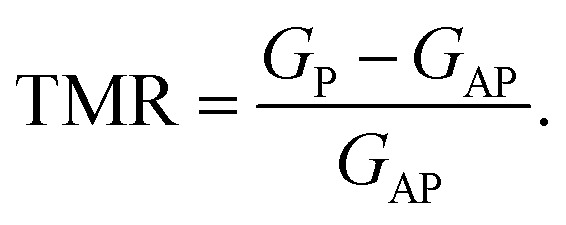


## 3 Results and discussion

### 3.1 Transmission mechanism of Ni/hBN/Ni MTJs with different numbers of hBN layers

The pd-hybridizations at both the upper and lower interfaces between the Ni slabs and hBN stabilize the system. These relevant chemical properties between Ni and hBN are also the basis of the unique electron transmission phenomenon appearing in hBN-based junctions. The evidence supporting this idea is presented in the following.

**Fig. 2:**
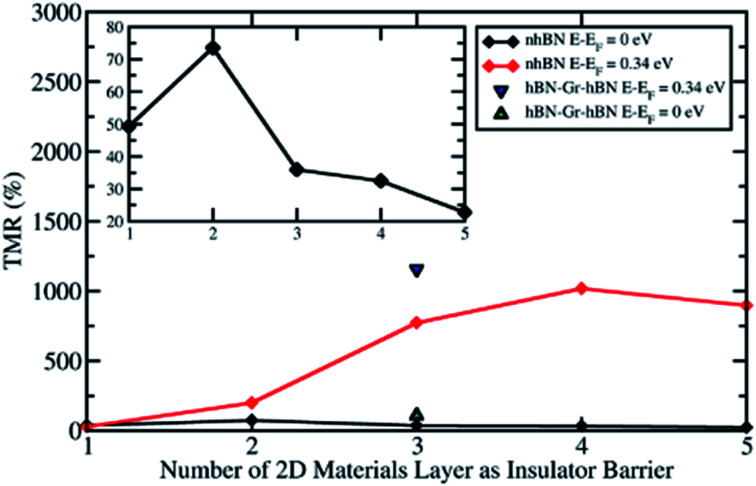
The TMR ratio *vs.* the number of 2D material layers as a tunnel barrier at two different energies; *E* − *E*_F_ = 0 eV (the zero-bias limit) and 0.34 eV (the highest peak of spin-down electron transmission probability in the PC state started from *n* = 3).

Fig. 2 shows the TMR ratio of 2D material based MTJs as a function of the number of 2D material layers as an insulator barrier. The black line shows the TMR ratio values of the Ni/*n*hBN/Ni MTJ, which was taken at the Fermi energy, namely the zero-bias limit, for various number of hBN layers, *i.e.*, *n* = 1–5. The TMR ratio profile is in good agreement with a previous theoretical study^45^ and is consistent with several experimental studies.^22^

When a double-hBN layer having two stacked hBN planes (2hBN) is considered as a tunnel barrier, the TMR ratio increases compared with a monolayer of hBN. Such an increase is due to a difference in the transmission process of the electrons. When a monolayer of hBN is considered as a tunnel barrier, due to pd-hybridizations from both upper and lower Ni slabs coupled with N atoms, charge transfer occurs, leading to the hBN layer becoming metallic.^31^ Thus, the propagating wave electrons become the dominant contribution for the transmission between the two Ni(111) electrodes.

**Fig. 3:**
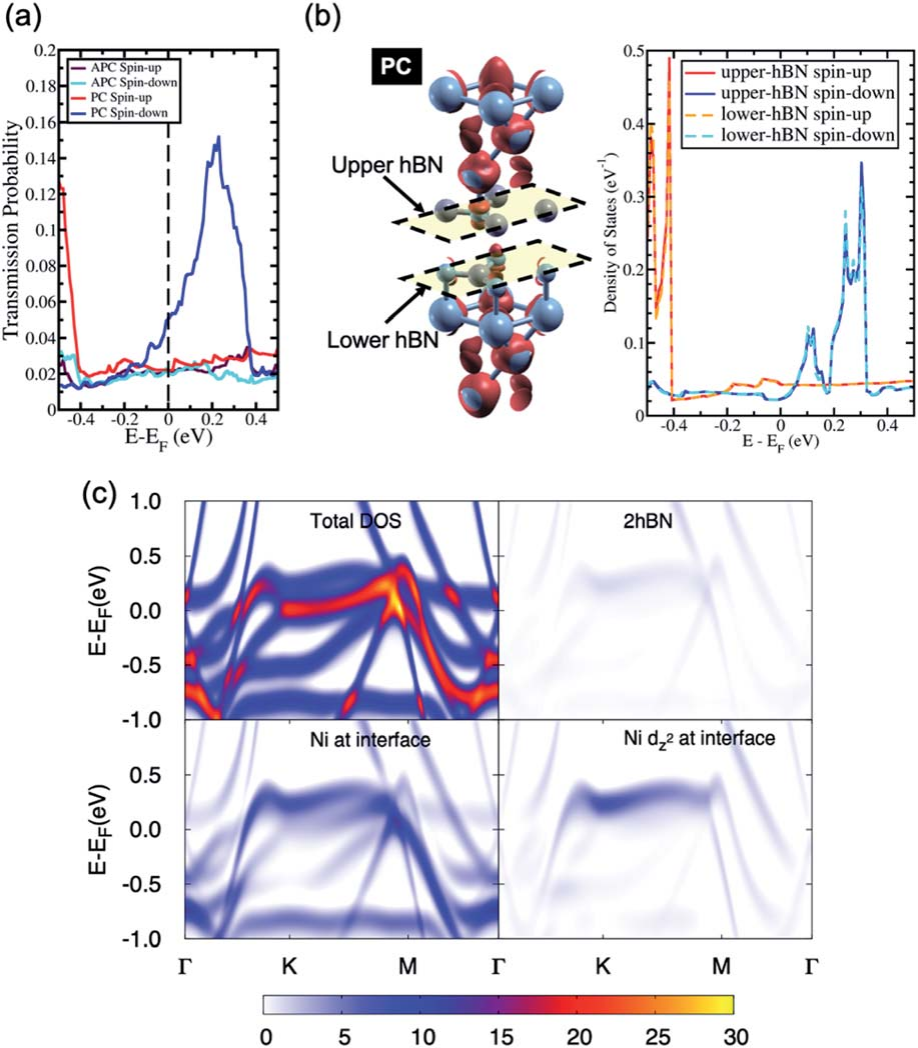
(a) The transmission probability of Ni/2hBN/Ni, (b) the spin-charge density mapping (the red color represents spin-up charge density mapping) and LDOS of the 2hBN insulator barrier in the Ni/2hBN/Ni system in the PC state, and (c) the projected bandstructure of Ni/2hBN/Ni for the spin minority channel.

On the other hand, when 2hBN is used as a tunnel barrier, due to the weak van der Waals interaction between the two hBN layers, electrons are transmitted through the tunnel barrier *via* the Ni(111) surface state at the interface. This transmission process can be observed from the transmission probability profile of the Ni/2hBN/Ni system in Fig. 3(a). In the Ni/hBN/Ni MTJ, the high transmission peak in the minority spin channel, observed when the system is in the PC state, is located at the Fermi energy when the Ni/hBN/Ni system is considered.^31^ However, in the Ni/2hBN/Ni MTJ, as shown in Fig. 3(a), the high transmission peak in the minority spin channel is shifted towards energy higher than Fermi energy by 0.24 eV.

On the other hand, the local density of states (LDOS) of hBN at the interfaces in Fig. 3(b) shows newly created states at the insulator gap of hBN. Furthermore, the LDOS of those states correlates with the profile of the transmission probability of the system, whereby an increase in the density of states at an energy higher than the Fermi energy in the spin minority channel corresponds to a high transmission peak in the spin minority channel. Therefore, these states represent the dominant contribution to the transmission of electrons through the insulator barrier.

The projected band structure in Fig. 3(c) shows that these states originate from the Ni(111) surface state with major d-components. The high transmission peak observed at an energy higher than the Fermi energy in the spin minority channel corresponds to the d_*z*^2^_-orbital of Ni(111) at the interfaces, which hybridizes with the p_*z*_-orbital of the N atoms. Simultaneously, the flat and broad states in Fig. 3(b) correspond to the s-orbital of Ni(111) at the interface. The relatively larger components in the d_*z*^2^_-orbitals compared with the p_*z*_-orbitals suggest that the wavefunction is indeed in a tunnel regime, where 2hBN behaves as a potential barrier for the Ni d-electrons.

**Fig. 4:**
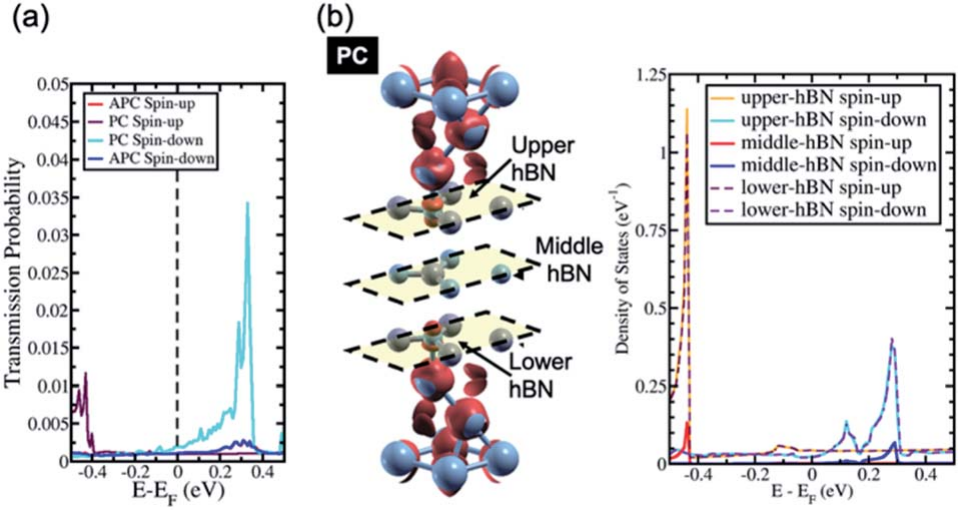
(a) The transmission probability of Ni/3hBN/Ni and (b) the spin charge density mapping of Ni/3hBN/Ni in the PC state (the red color represents spin-up charge density mapping) and LDOS of the 3hBN insulator barrier in the Ni/3hBN/Ni system in the PC state.

When the number of hBN layers is increased further, *e.g.*, 3hBN, the contribution from these surface states at the Fermi energy is quenched. As a result, the surface states of Ni(111), which are derived from the s-orbital of the system with a number of hBN layers higher than two, become weaker around the Fermi energy, as shown in Fig. 4(b). This quenching leads to a drop in the transmission probability of electrons in the spin minority channel of the PC state at Fermi energy, which becomes approximately equivalent to the transmission probability of electrons in the APC state as shown in Fig. 4(a). This result reduces the TMR ratio of the system at Fermi energy, which becomes lower than that observed for Ni/2hBN/Ni. Furthermore, when the number of hBN layers is further increased to 4hBN and 5hBN, a monotonic decrease in the TMR ratio with increasing number of hBN layers is expected just around the Fermi energy.

### 3.2 High transmission magnetoresistance on the excited state and influence of the proximity effect

**Fig. 5:**
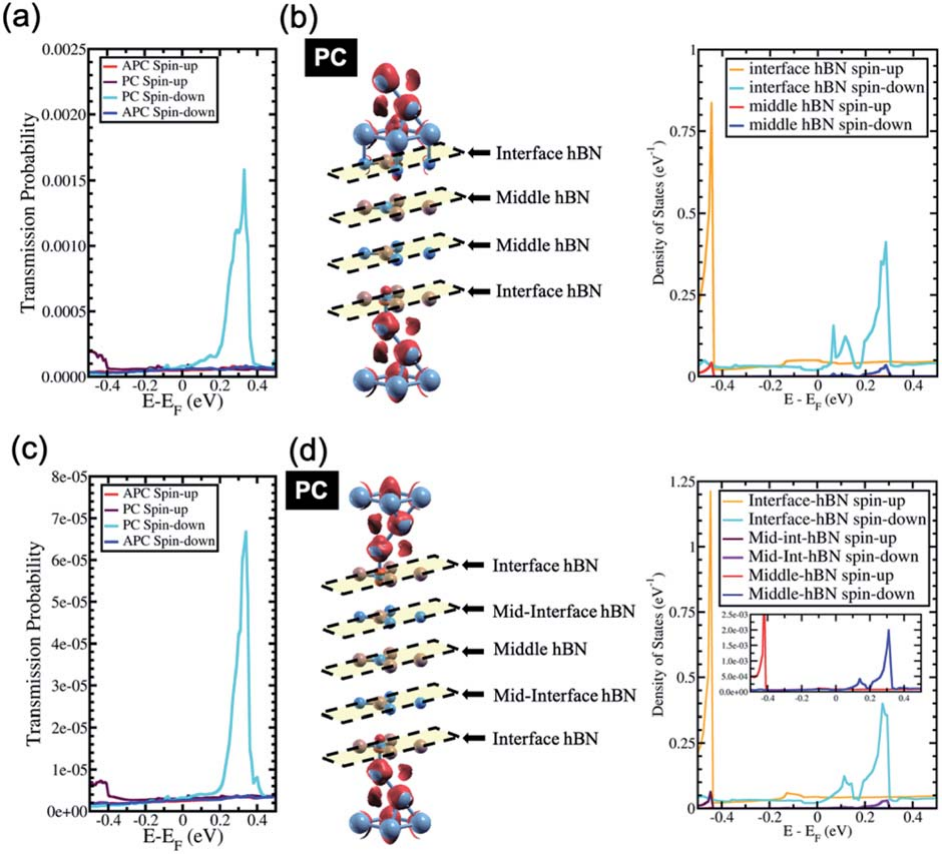
(a) The transmission probability of Ni/4hBN/Ni, (b) the charge density mapping of Ni/4hBN/Ni (the red color represents spin-up charge density mapping) and the LDOS of the 4hBN insulator barrier of the Ni/4hBN/Ni system in the PC state, (c) the transmission probability of Ni/5hBN/Ni, and (d) the charge density mapping of Ni/5hBN/Ni (the red color represents spin-up charge density mapping) and the LDOS of the 5hBN insulator barrier of the Ni/5hBN/Ni system in the PC state (inset: magnified LDOS of the middle hBN of the Ni/5hBN/Ni system).

In the last section, the presence of a peak in the transmission probability was discussed. As shown in Fig. 3 and 4, the high transmission peak of electrons in the spin minority channel, when the MTJ is in the PC state, was observed at energy slightly higher than Fermi energy (*E* − *E*_F_ = 0.24 eV for Ni/2hBN/Ni and *E* − *E*_F_ = 0.34 eV for Ni/3hBN/Ni). Interestingly, this high transmission peak of electrons is still present upon increasing the number of hBN layers. From the transmission probability of electrons of Ni/*n*hBN/Ni with *n* = 4 and 5, the peak of the high transmission probability of electrons in the spin minority channel, when the MTJ is in the PC state, was also observed at energy 0.34 eV higher than the Fermi energy, as shown in Fig. 5(a) and (c), which is the same as that for Ni/3hBN/Ni. Interestingly, it was found that at *E* − *E*_F_ = 0.34 eV for Ni/*n*hBN/Ni with *n* = 1–4, a high and increasing TMR ratio is observed but decreases when *n* = 5, as shown by a red line in Fig. 2. To understand this behavior, a further investigation was done on the electronic state of the insulator barrier for the Ni/*n*hBN/Ni system with *n* = 4 and 5.

The LDOS of the hBN layer in the insulator barrier for the Ni/*n*hBN/Ni system with *n* = 4 and 5 is shown in Fig. 5(b) and (d), respectively. Looking at the LDOS of hBN in the insulator barrier for *n* = 3, 4, and 5, a typical behavior corresponding to the presence of the magnetic proximity effect, which is the penetration depth of the spin polarization of a magnetic metal (Ni) into a nonmagnetic material (hBN), is observed. This proximity effect, which causes an evanescent wave, or a damping mode inside hBN, is found as the LDOS of the hBN layer, at *E* − *E*_F_ = 0.34 eV, gradually decreases when hBN moves far from the interface. However, the LDOS still survives at *E* − *E*_F_ = 0.34 eV even for the middle hBN in the Ni/5hBN/Ni system. A sharp damping is observed because the proximity effect of d_*z*^2^_ acts on the p_*z*_ orbital of B, which is unoccupied. At energy close to the Fermi energy, since the energy gap of hBN does exist, no real propagating modes in the hBN slab are expected. On the other hand, at *E* − *E*_F_ = 0.34 eV, since a small LDOS is still observed through all of the hBN in the tunnel barrier, propagating modes in the hBN slab are expected. Thus, for the case of Ni/*n*hBN/Ni with *n* > 2, the proximity effect becomes the main contribution for the transmission of the electron with the energy being 0.34 eV higher than the Fermi energy, through the hBN tunnel barrier.

This tunneling behavior causes an interesting filtering effect. In the PC state, when *E* − *E*_F_ = 0.34 eV, the minor-spin channel exhibits a high transmission probability. In contrast, for the major-spin channel, the transmission is significantly reduced. The high transmission for the minor-spin channel originates from the d-orbital nature since a large pd-hybridization causes a spin-split LDOS with a prominent peak at the corresponding energy. Thus, except for the d channel, a lowered transmission can only be expected. Therefore, the major-spin component shows a reduced transmission. As a result, the ratio of the minority-spin transmission and majority-spin transmission should become more prominent as *n* increases. This high transmission probability is found only when a parallel-spin configuration is selected with the pd-hybridization occurring on both sides of hBN.

In the case of the APC state, a blocking behavior can be observed. An optimum blocking behavior was observed when 4hBN is used as an insulator barrier. On Ni/3hBN/Ni, in the APC state, spin majority and minority channels show a small but non-negligible transmission probability at *E* − *E*_F_ = 0.34 eV. This result follows from the fact that the middle hBN is not spin-polarized due to an opposite proximity effect acting on it from lower and upper Ni/hBN interfaces. Thus, it makes the blocking behavior imperfect and causes a small number of electrons to pass through the insulator barrier for spin minority and majority. However, for Ni/4hBN/Ni, the two middle hBN layers have opposite spin-polarization to each other, making the blocking behavior much stronger and resulting in lower electron transmission probability for both spin majority and minority. By comparing these low transmission probability in APC state with the high transmission of electrons in the spin minority channel while the system is in PC state; as a result, the TMR ratio at *E* − *E*_F_ = 0.34 eV becomes optimum.

When increasing further the thickness of hBN layers as an insulator barrier into 5hBN, the TMR ratio at *E* − *E*_F_ = 0.34 eV is reduced, as shown in the red line in Fig. 2. This behavior arises because the proximity effect found in the middle hBN becomes weaker, although the blocking effect has become stronger since 4hBN was used as an insulator barrier. This fact is clearly shown in Fig. 5(d), where the LDOS of the middle hBN at *E* − *E*_F_ = 0.34 eV becomes much smaller compared to those of the middle-interface and interface hBN. This smaller LDOS causes the peak of high transmission of the minority spin electron in the PC state to reduce significantly compared to that of the Ni/4hBN/Ni system. Therefore, the TMR ratio is reduced and becomes smaller than that of the Ni/4hBN/Ni system.

**Fig. 6:**
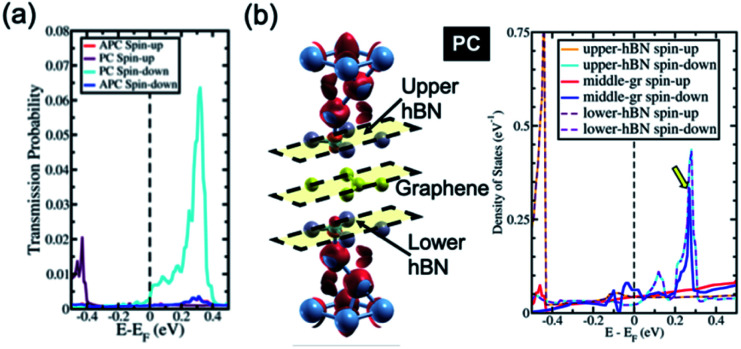
(a) The transmission probability of Ni/hBN–Gr–hBN/Ni and (b) the spin charge density mapping of Ni/hBN–Gr–hBN/Ni (the red color represents spin-up charge density mapping) and LDOS of the hBN–Gr–hBN insulator barrier of the Ni/hBN–Gr–hBN/Ni system in the PC state.

Interestingly, the proximity effect can be magnified when the hBN layer is replaced with the graphene layer, for example, replacing the unhybridized hBN layer of the Ni/3hBN/Ni MTJ with graphene, thus yielding Ni/hBN–Gr–hBN/Ni, as shown in Fig. 6(b). The interface connection from Ni to hBN was very similar between Ni/3hBN/Ni and Ni/hBN–Gr–hBN/Ni junctions since the Ni–N interlayer distance is 2.05 Å for both junctions. Fig. 6(b) also shows the LDOS of the hBN–Gr–hBN insulator barrier. The LDOS of hBN at the interface in Fig. 6(b) shows a very similar value to the LDOS of the upper (or lower) hBN in Ni/3hBN/Ni. However, there is a clear difference in LDOS on graphene from the middle hBN of Ni/3hBN/Ni. Fig. 6(b) shows an approximately 6-times higher electronic density of states on graphene at *E* − *E*_F_ = 0.34 eV compared to the center hBN in the Ni/3hBN/Ni MTJ for the spin-down channel. Thus, these states lead to a higher transmission probability at the corresponding energy. As shown in Fig. 6(a), a high magnetoresistance ratio of ∼1200% can be observed for the Ni/hBN–Gr–hBN/Ni MTJ at *E* − *E*_F_ = 0.34 eV. This TMR ratio is higher than that of the Ni/4hBN/Ni MTJ, which has the highest TMR ratio among Ni/*n*hBN/Ni MTJs, as shown in Fig. 2. The high performance and unique characteristics of the Ni/hBN–Gr–hBN/Ni MTJ could provide novel functionalities, such as optically induced MTJs, which are introduced in the following section.

### 3.3 Proposed design and mechanism of optically induced MTJs

**Fig. 7:**
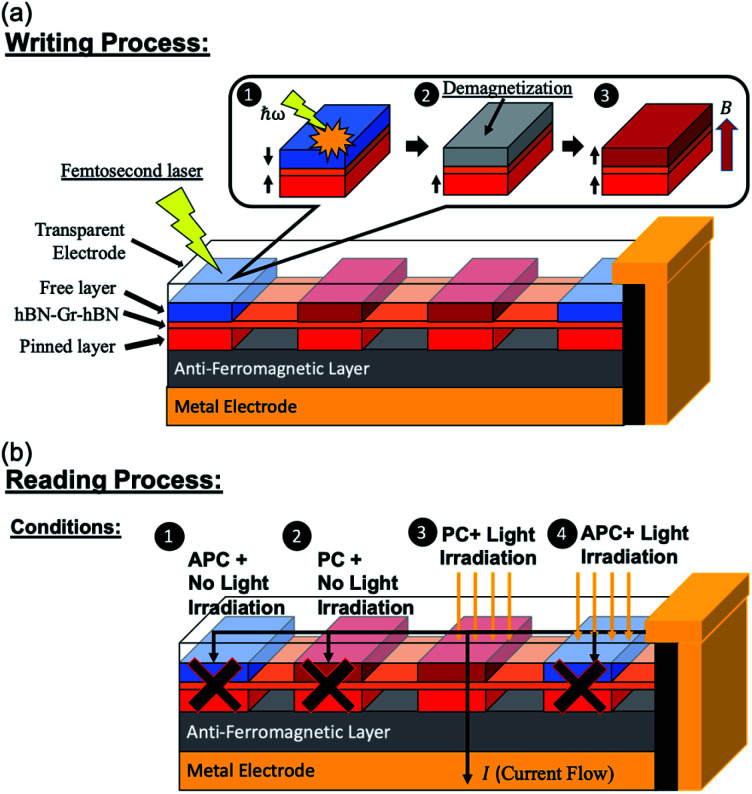
(a) The writing and (b) the reading process of the proposed Ni/hBN–Gr–hBN/Ni MTJ device with light irradiation.

The proposed idea of using a Ni/hBN–Gr–hBN/Ni MTJ as an optically induced MTJ is shown in Fig. 7. The process of reading and writing data in a memory using light irradiation is considered here. For the writing process, as discussed in a previous study, an optical demagnetization using a circularly polarized femtosecond or sub-picosecond laser and field-assisted magnetic switching can change the magnetic orientation of the free Ni(111) ferromagnetic layer.^46^ The high performance and unique characteristics of the Ni/hBN–Gr–hBN/Ni MTJ are primarily used for the reading process. Two Au electrodes were used to make the current flow into the proposed MTJ device. A small bias voltage was applied. At first, the current flows from the Au electrode to the transparent electrode on the top of the Ni/hBN–Gr–hBN/Ni MTJ. Without any light irradiation onto the system, the reading process cannot be optimally carried out since the current passing through the MTJ is relatively small in both APC and PC states. The small current is observed because, at Fermi energy, the electron transmission for the APC and PC states is low, as explained in the previous section. Reading conditions 1 and 2 are illustrated in Fig. 7(b). When linearly polarized light, *e.g.*, infrared or visible light, is used to excite the electrons below the Fermi energy to an energy 0.34 eV higher than the Fermi energy from the upper Ni slabs, the optimal reading process is observed. Condition 3, illustrated in Fig. 7(b), shows that a high transmission occurs in the PC state resulting in the flow of current through the MTJ towards the lower Au electrode. In addition, condition 4 shows that the MTJ is in the APC state, thus explaining the observed low transmission.

## 4 Conclusions

An investigation into Ni/*n*hBN/Ni MTJs was conducted by increasing the number of hBN layers in the tunnel barrier. Owing to the electrons' transmission through the surface states, an increasing TMR ratio was observed when considering 2hBN as the tunnel barrier compared with a monolayer hBN. However, a monotonic decrease in TMR ratio was found when more than two hBN layers were considered. This behavior is due to the quenched surface states of Ni(111) at Fermi energy, observed at the insulator gap of hBN.

On the other hand, it was found that the Ni/*n*hBN/Ni MTJ exhibits a slight shift of the highest transmission peak towards energy higher than the Fermi energy. This result follows from the electron transmission through Ni d_*z*^2^_ which hybridized with N p_*z*_. Interestingly, a high and increasing TMR ratio was observed at an energy where the high transmission peak is located. These results are due to the proximity effect of the unhybridized hBN layer. The effect of the proximity can be magnified when the hBN layer is replaced with the graphene layer. When Ni/3hBN/Ni is considered, and the unhybridized hBN layer in Ni/3hBN/Ni is replaced with a graphene layer, the stronger proximity effect becomes evident through a higher electronic density of states at the corresponding energy for the LDOS of graphene. Thus, these states lead to a higher transmission probability at the corresponding energy. A high magnetoresistance ratio of ∼1200% was observed for the Ni/hBN–Gr–hBN/Ni MTJ at *E* − *E*_F_ = 0.34 eV.

This Ni/hBN–Gr–hBN/Ni MTJ's high performance and unique characteristics allow the possibility to exploit a novel device functionality, namely, that of an optically induced MTJ. The process of reading and writing in this proposed MTJ is expected to be conducted by light irradiation. Optical demagnetization and field-assisted magnetic switching are expected to change the magnetic orientation of the free Ni(111) ferromagnetic layer. This process would represent the writing process in the proposed device. On the other hand, the unique characteristics of the Ni/hBN–Gr–hBN/Ni MTJ would mainly contribute to the reading process. The linear polarization of light was applied to induce the transmission to occur at energy higher than Fermi energy by 0.34 eV. This process was exploited to read the magnetic alignment of the Ni/hBN–Gr–hBN/Ni MTJ.

## Author contributions

Conceptualization was done by H. H. and Y. W. H. H. studied the transmission probability and found high TMR ratio at energy higher than Fermi energy. The charge density mapping was performed by H. H. Y. W. examined the LDOS and projected band structure to understand the transmission mechanism. The new writing and reading process mechanism was proposed by Y. W. K. K. performed supervision on the research and proposed a detailed explanation for the proximity effect. Y. W. and H. H. wrote the original draft of the manuscript. K. K., G. K. S., and M. A. M. wrote and edited the manuscript. All the authors reviewed the manuscript.

## Conflicts of interest

There are no conflicts to declare.

## Supplementary Material

NA-004-D1NA00272D-s001
