# Supplementary material for: High magnetoresistance of a hexagonal boron nitride–graphene heterostructure-based MTJ through excited-electron transmission
Source: Nanoscale Adv. 2021 Oct 21;4(1):117–24. doi: 10.1039/d1na00272d (PMC9417163; doi:10.1039/d1na00272d)

Electronic Supplementary Material (ESI) for Nanoscale Advances.
This journal is © The Royal Society of Chemistry 2021

Electronic Supplementary Information

High magnetoresistance of hexagonal boron nitride- graphene heterostructure-based MTJ through excited-electron transmission

Halimah Harfah,*[a‡] Yusuf Wicaksono,[a‡] Gagus Ketut Sunnardianto,[c] Muhammad Aziz Majidi,[b] and Koichi Kusakabe[a,d]

a. *Graduate School of Engineering Science, Osaka University, 1-3 Machikaneyama-cho, Toyonaka, Osaka 560-8531, Japan.*

b. *Department of Physics, Faculty of Mathematics and Natural Science, Universitas Indonesia, Kampus UI Depok, Depok, Jawa Barat 16424, Indonesia.*

c. *Research Center for Physics, The National Research and Innovation Agency, Kawasan Puspiptek Serpong, Tangerang Selatan, 15314, Banten, Indonesia.*

d. *School of Science, Graduate School of Science, University of Hyogo, 3-2-1 Kouto, Kamigori-cho, Ako-gun, Hyogo, 678-1297, Japan.*

‡ These authors contributed equally to this work

* Corresponding author; E-mail: harfah.h@opt.mp.es.osaka-u.ac.jp

Table S1. *The comparison between various van der Waals interactions method in DFT calculation on Ni/3hBN/Ni MTJ respect to experimental result on epitaxial hBN placed on Ni(111) surface [1] and bulk hBN [2]. DFT-D3 and DFT-D2 show an interlayer distance between Ni-hBN and hBN-hBN closer to experimental values.*

	Ni-hBN Layer Distance	hBN-hBN layer Distance
Experimental	1.87 ± 0.12 Å [1]	3.33 Å [2]
DFT-D2 method	2.07 Å (N-Ni)<br>1.95 Å (B-Ni)	3.44 Å (N-B)<br>3.56 Å (B-N)
DFT-D3 method	2.05 Å (N-Ni)<br>1.93 Å (B-Ni)	3.13 Å (N-B)<br>3.25 Å (B-N)
vdW-DF method	3.52 Å (N-Ni)<br>3.52 Å (B-Ni)	3.53 Å (N-B & B-N)
vdW-DF2 method	3.56 Å (N-Ni)<br>3.56 Å (B-Ni)	3.48 Å (N-B & B-N)

[1] Tonkikh, A. A. et. al., Structural and electronic properties of epitaxial multilayer h-BN on Ni(111) for spintronics applications. *Sci. Rep.* **6**, 23547 (2016).

[2] Lynch, R. W. Effect of high pressure on the lattice parameters of diamond, graphite, and hexagonal boron nitride. *J. Chem. Phys.* **44**, 181 (1966).

Figure S1. *The transmission probability of Ni/3hBN/Ni and Ni/hBN-vacuum-hBN/Ni in PC state for spin-up and spin-down electrons. The hBN at the interfaces distance of Ni/hBN-vacuum-hBN/Ni MTJ was made same with Ni/3hBN/Ni MTJ. This result shows that the wide-gap insulator of hBN can never be treated as a vacuum for the conducting electron. This result also indicates that the high transmission probability peak of the spin-down electron in the PC state found in the 3hBN, 4hBN, and 5hBN system comes from the proximity effect, which works on middle hBN.*

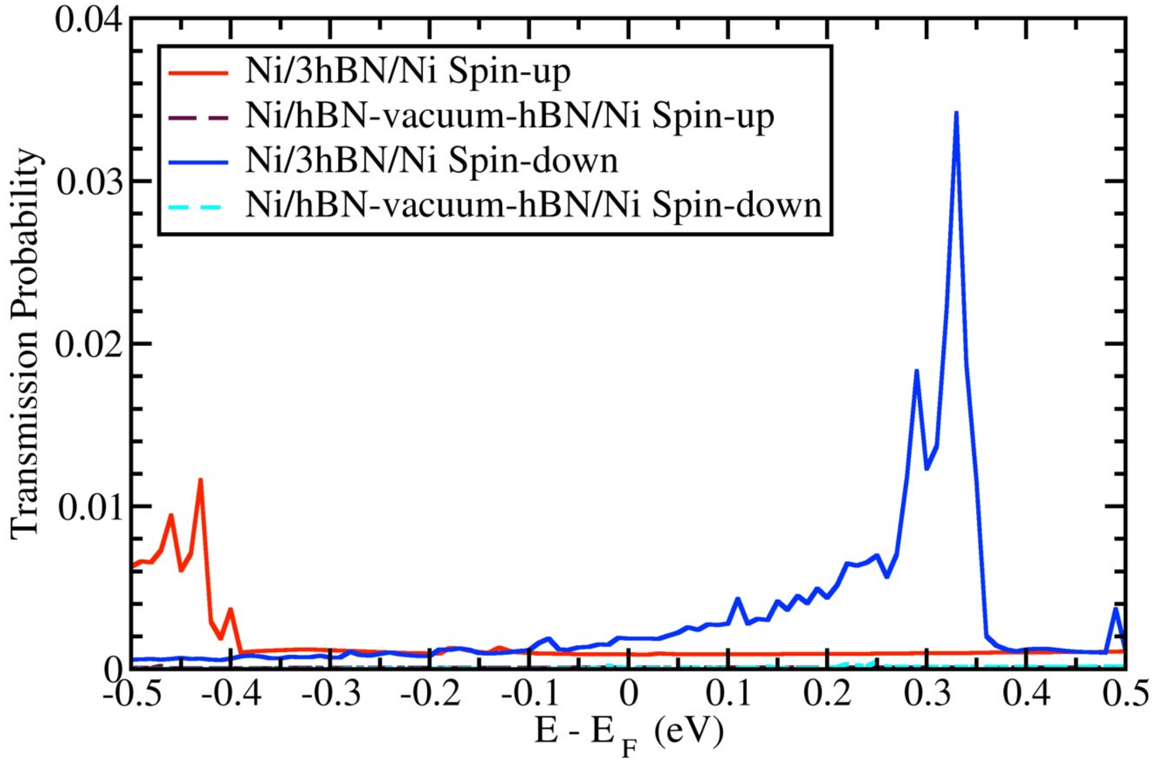

Figure S2. *The local density of states (LDOS) of insulator barrier of (a) Ni/3hBN/Ni and (b) Ni4hBNNi (only middle hBN shows in the graph) when upper and lower Ni slabs in anti-parallel configuration (APC).*

(a)

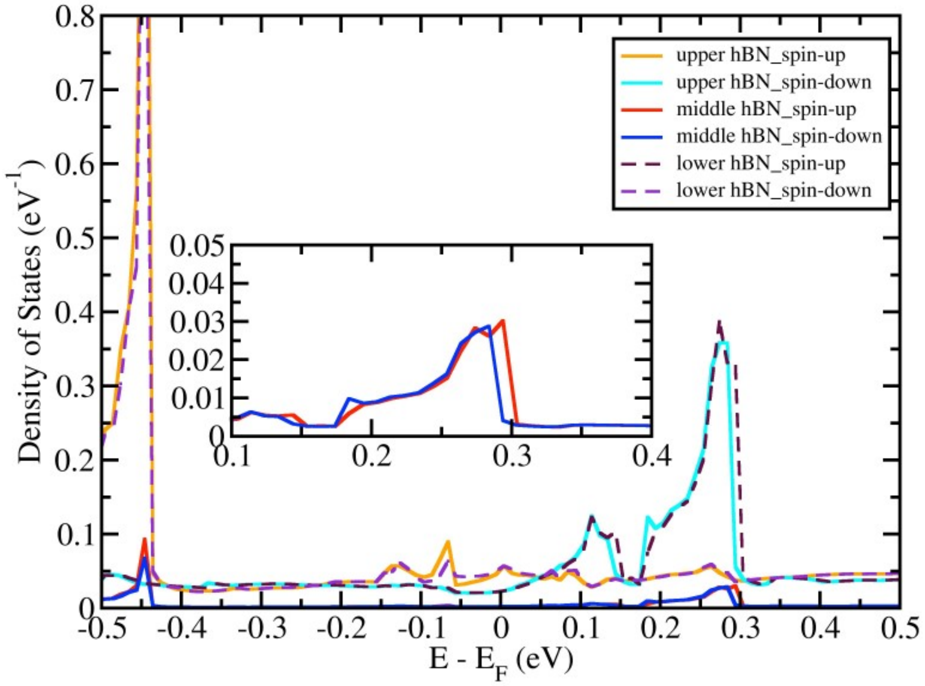

(b)

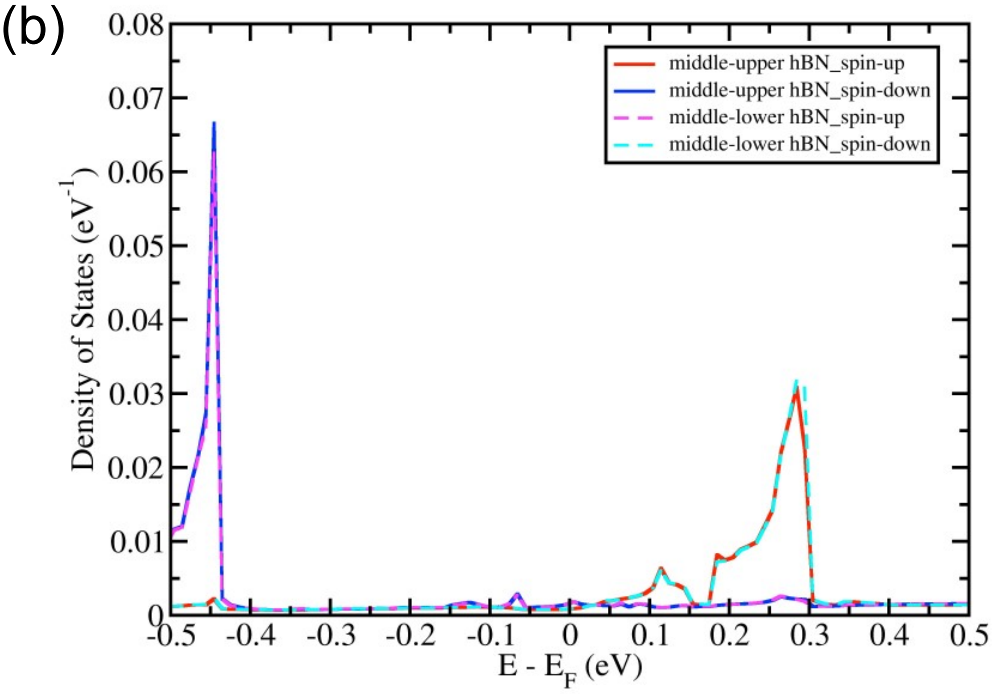

Supplement: NA-004-D1NA00272D-s001 [file NA-004-D1NA00272D-s001.pdf]
